# A preliminary study of calcium channel-associated mRNA and miRNA networks in post-traumatic epileptic rats

**DOI:** 10.1038/s41598-023-39485-9

**Published:** 2023-08-11

**Authors:** Xiao Jia, Yixun Ma, Xiaoyuan Zhang, Zefang Shen, Min Wang, Lufang Jiang, Xuan Wei, Chang Li, Mengzhou Zhang, Tiantong Yang

**Affiliations:** 1 Key Laboratory of Evidence Science (China University of Political Science and Law), Ministry of Education, No. 25 Xitucheng Road, Haidian District, Beijing, 100088 China; 2 Collaborative Innovation Center of Judicial Civilization, Beijing, 100088 China; 3 State Key Laboratory of Medicinal Chemical Biology and College of Pharmacy, Nankai University, Tianjin, 300350 China; 4 College of Biological Science, China Agricultural University, Beijing, 100193 China; 5 Chinese Institute for Brain Research, Beijing, 102206 China

**Keywords:** Molecular biology, Neuroscience, Biomarkers, Medical research, Molecular medicine, Neurology

## Abstract

The calcium channels are the main pathogenesis and therapeutic target for post-traumatic epilepsy (PTE). However, differentially expressed miRNAs (DEMs) and mRNAs associated with calcium channels in PTE and their interactions are poorly understood. We produced a PTE model in rats and conducted RNA-seq in PTE rats. Gene annotation was used to verify differentially expressed mRNAs related to calcium channels. RNAhybrid, PITA, and Miranda prediction were used to build the miRNA–mRNA pairs. Furthermore, Gene ontology (GO) and Kyoto Encyclopedia of Genes and Genomes (KEGG) pathway analysis were used for the functional enrichment analysis of DEMs. The quantification changes of mRNA and miRNA were verified by RT-qPCR. There were 431 identified differentially expressed genes (DEGs) in PTE rats compared with the sham group, of which five mRNAs and 7 miRNAs were related to calcium channels. The miRNA–mRNA network suggested a negative correlation between 11 pairs of miRNA–mRNA involved in the p53 signaling pathway, HIF-1 signaling pathway. RT-qPCR verified three upregulated mRNAs in PTE rats, associated with 7 DEMs negatively related to them, respectively. This study has revealed the changes in miRNA–mRNA pairs associated with calcium channels in PTE, which might contribute to the further interpretation of potential underlying molecular mechanisms of PTE and the discovery of promising diagnostics.

## Introduction

Epilepsy, characterised by recurrent seizures, is a common neurological disease^1^. The incidence of a single seizure during one’s lifetime has been reported to be approximately 10%. One-third of those who experience one seizure is diagnosed with "epilepsy"^2^. Post-traumatic epilepsy (PTE), also known as "acquired epilepsy", is a kind of epilepsy following traumatic brain injury (TBI) that tends to occur in about 20% of all epileptic patients^3^. Until now, the pathogenetic mechanisms of PTE have remained largely unknown. And prevention, diagnosis, and treatment of PTE are inferred as key difficulties in neuroscience^4^.

The calcium channels are closely relevant to the pathogenesis of PTE, such as abnormal neuronal discharge, glutamate release, neuronal apoptosis, glial scar hyperplasia, and synaptic remodeling^5^. The voltage-gated calcium (Ca^2+^) channels are divided into low-voltage (LVA) and high-voltage activated (HVA) channels. The T-type Ca^2+^ channels include CaV3.1–CaV3.3, while the HVA channels include CaV1 (L-type), CaV2.1 (P/Q-type), Cav2.2 (N-type) and Cav2.3 (R-type) channels. HVA channels have a high depolarization threshold and are responsible for neurotransmitter release from presynaptic terminals, while LVA channels are more frequently expressed in neuronal bodies and dendrites and are associated with the development of athetoid epilepsy and TLE. Changes in intracellular Ca^2+^ levels and Ca^2+^ homeostasis mechanisms are associated with seizures and acquired electrogenesis. Following acute epileptogenic brain injury, there is an irreversible increase in Ca^2+^ leading to neuronal death, followed by a sub-lethal, prolonged, but reversible increase in Ca^2+^ levels, triggering pathological changes that in turn lead to the development of PTE^4^. More importantly, calcium channel blockers (e.g., phenytoin sodium and levetiracetam) have long been the preferred drugs in clinical anti-epileptic treatment^6^. Nevertheless, these are ineffective in some patients, which might be due to an undefined molecular regulatory mechanism of calcium channels in PTE^6^. Accordingly, it is important to investigate calcium regulatory mechanism interactions in PTE related to calcium channels.

Genes affect biological functions (e.g., ionic equilibrium and apoptosis) by regulating the proteins they encode, while abnormal expression of the protein encoded by the gene often leads to diseases. Furthermore, the interplay between coding and non-coding RNAs has always been the core of gene expression^7^. MiRNA, a single-stranded non-coding RNA about 21–23 nucleotides in length, regulates gene translation by specifically combining with the 3′ end of its target mRNA^8^. MiRNAs can regulate about 60% of human mRNAs and extensively participate in biological processes, such as cell apoptosis, synaptic remodeling, and neural development^9^. Differentially expressed miRNAs (DEMs) and mRNAs can alter various cellular and biological functions, leading to diseases. Over recent years, it has been suggested that an integrated analysis of miRNA and mRNA networks was beneficial to understand the potential molecular mechanisms of cancers and ageing^7,10^. Nevertheless, the miRNAs and mRNAs interaction network associated with the calcium channels in PTE was poorly understood and needed to be comprehensively analysed and elucidated.

In this study, we integrated and analyzed the miRNA and mRNA expression profiling transcriptome in PTE rats to clarify which mRNA and miRNA might be related to calcium channels in PTE. We then constructed a calcium channel-related mRNA–miRNA regulatory network in PTE and further confirmed the negative correlation. Our study revealed that five mRNAs and 7 miRNAs are associated with calcium channels. The miRNA–mRNA network suggested a negative correlation between eleven pairs of miRNA–mRNA involved in the p53 signaling pathway, HIF-1 signaling pathway. RT-qPCR was verified on mRNAs and miRNAs in PTE rats, respectively.

## Materials and methods

### Animal model

#### Animal housing and care

Fifteen male Sprague–Dawley rats weighing 210–230 g (Beijing Laboratory Animal Research Center, Beijing, China) were used in our model. In the sterile environment, all the rats were kept on a 12 h/12 h light/dark cycle and provided free food and water access.

#### Animal surgical procedure

After 1 week of adaptive feeding, the rats were assigned to either a sham or a PTE group. There were six rats in the sham-operated group and six rats in the PTE group. The modelling of PTE was established according to our previous study^11^. Briefly, the rats were anaesthetized with 2% pentobarbital sodium (40 mg/kg) and placed in a stereotactic device (Nanjing Med ease Science and Technology, Jiangsu, China). The rat skull was drilled to expose the injection target (2.0 mm anterior to bregma, 3.0 mm from the midline, and 2.0 mm depth). The PTE group was injected with 10 µl of 0.1 mol/l FeCl_2_ through the injection target at a rate of 1 µl/min, while the sham group underwent the same procedures except for the injection of FeCl_2_.

#### Animal post-surgery care

Rats were continuously observed for 1–2 h following surgery. Once injected, the behaviour of rats was observed and marked according to the Racine grading^12,13^. EEG monitoring was performed by electrodes implanted in the frontal and occipital lobes of rats, along with a bio signal acquisition system (Shanghai Creaform3D Information, Shanghai, China). The 1-h EEG, including the first 15 min and subsequent 10 min at 5 min intervals, was documented before operation and 1 h, 1 day, 7 days, and 30 days post-operation. The criteria for the successful establishment of the PTE rat model are as follows: (1) Racine's score greater than 4. And the EEG test result with sharp waves or spikes or paroxysmal appearance or persistent abnormal discharge was considered successful modelling of PTE rats. Finally, we euthanized the rats using carbon dioxide and extracted brain tissue for subsequent experiments.

### RNA extraction

The frontal brain tissues from the FeCl_2_ injection region of rats were gathered, of which total RNA was extracted using TRIzol™ Reagent (Thermo Fisher SCIENTIFIC, Product No.: 15596026) and was transcribed into cDNA according to the manufacturer’s instruction. The purity and integrity of isolated total RNA were analyzed by a Nanodrop 2000 spectrophotometer (IMPLEN, CA, USA) and a Bioanalyzer 2100 system (Life Technologies, Carlsbad, CA, USA). We used the following methods to ensure the purity, integrity, and concentration of the extracted RNA samples: (1) agarose gel electrophoresis to analyses the purity and integrity of the RNA; (2) nanodrop to detect the purity of the RNA (OD 260/280 ratio); (3) qubit 2.0 to accurately quantify the concentration of RNA; (4) agilent 2100 to accurately detect the integrity of the RNA.

### Preparation of the sequencing library and RNA-seq

Libraries for sequencing were constructed using 3 μg RNA of each RNA sample according to the manufacturer's instructions. After the RNA samples have been tested, the Ribo-Zero™ kit is generally used to remove rRNA from the total RNA samples (some lncRNAs have the same polyA tail structure as mRNAs, and rRNA removal maximises the retention of lncRNAs with polyA tails). Add fragmentation buffer to the enriched RNA to break the RNA into small fragments. The fragmented RNA was then used as a template to synthesise the first strand of cDNA by adding 6 bp random hexamers, and the second strand of cDNA was synthesized with the addition of buffer, dNTPs (dTTP was replaced by dUTP), DNA polymerase I and RNase H. The cDNA was then synthesized into a double-stranded cDNA by adding 6 bp random hexamers to the buffer and dNTPs. The synthesized double-stranded cDNA was purified, end-repaired, A-added and ligated into sequencing junctions. The U-containing second strand of the cDNA was degraded by the USER enzyme and enriched by PCR, and the PCR products were purified by AMPure XP beads to obtain the final strand-specific library. The final strand-specific libraries were purified from PCR products using AMPure XP beads. After the libraries were constructed, Qubit 2.0 was used for preliminary quantification and dilution of the libraries, and then an Agilent 2100 (Agilent Technologies, CA, USA) was used to check the insert sizes of the libraries, and after the inserts matched the expected size, the effective concentration of the libraries was accurately quantified using the Q-PCR method to guarantee the quality of the libraries. Finally, the 150-bp paired-end reads and the 50-bp single-end reads were generated for mRNA and miRNA sequencing, respectively, using the Illumina HiSeq 2500 sequencer.

### Bioinformatics analysis

Raw expression data sets of mRNA and miRNA were obtained from RNA-seq. We first eliminated reads containing adapters or multiple Ns and low-quality reads from the raw reads (FASTQ format) to obtain clean reads. The clean reads were then used to match the reference genome (ratrelease91) downloaded from the Ensembl database using Bowtie v2.2.3 and TopHat v2.0.12, and then the transcript fragments per kilobase per million mapped reads were calculated for each transcript. miRNA expression was compared to the expression of miRNA precursors and corresponding mature miRNAs in miRbasev22 using miRDeep2.According to our previous research^12^, these data were preprocessed by background correction and normalization using the DESeq2 R package. Differentially expressed genes (DEGs) were identified when their corrected *P* value < 0.05 according to the Benjamini–Hochberg method. All DEGs were analyzed with the R/Bioconductor ggplot2 package. The miRNA–mRNA networks were built by RNAhybrid, PITA, and Miranda methods^14^. The Gene ontology (GO) and Kyoto Encyclopedia of Genes and Genomes (KEGG) pathway analyses were conducted on the DEMs using the R/Bioconductor cluster Profiler and Reactome PA package.

### Gene expression assessment

Total RNA extraction and reverse-transcription quantitative polymerase chain reaction (RT-qPCR) were conducted as detailed in our previous studies^15^. Primer sequences of mRNA and miRNA are listed in Tables 1 and 2, respectively. The housekeeping gene GAPDH and U6 snRNA were amplified as an internal control. The data were analyzed using the 2^ΔΔ^CT comparative method.

**Table 1:** Primer sequences of mRNA for RT-qPCR.

Primer name	Primer sequence
GAPDH qPCR-F	5′-CACCAGCATCACCCCATT-3′
GAPDH qPCR-R	5′-CCATCAAGGACCCCTTCATT-3′
CALU qPCR-F	5′-CAGACTATGACCATGCAGAGGC-3′
CALU qPCR-R	5′-AGAACTCATCGTGTCGTACTAAGG-3′
CAMK2B qPCR-F	5′-CTCACACAGTACATCGACGGCC-3′
CAMK2B qPCR-R	5′-CCGAGCAGTGGAAATGGACATT-3′
Lrguk qPCR-F	5′-CTTAAAAGCACTTCGTGTAACATCC-3′
Lrguk qPCR-R	5′-CTATCTTCGGCCATGTGGGATTG-3′

**Table 2:** Primer sequences of miRNA for RT-qPCR.

MiRNA	Primer sequence
miR-98-5p	5′-AACAATACAACTTACTACCTCA-3′
miR-103-3p	5′-TCATAGCCCTGTACAATGCTGCT-3′
miR-30e-5p	5′-CTTCCAGTCAAGGATGTTTACA-3′
miR-335	5′-ACATTTTTCGTTATTGCTCTTGA-3′
miR-449a-5p	5′-ACCAGCTAACAATACACTGCCA-3′
miR-139-5p	5′-CTCCAACAGGGCCGCGTCTCCA-3′
miR-138-5p	5′-CGGCCTGATTCACAACACCAGCT-3′
U6	5′-TTGCGTGTCATCCTTGCGCAGG-3′

### Statistical analysis

RT-qPCR values were expressed as mean ± SD. Analysis of variance (ANOVA) and Student's two-tailed unpaired t-test was used to measure the difference between the mean RT-qPCR values of the two groups in the PTE and shame groups. Statistical analysis and graph production were performed with SPSS 25.0 (IBM, Armonk, NY, USA) and GraphPad Prism 8 (San Diego, CA, USA), respectively. The *P* values < 0.05 was considered statistically significant.

### Ethical approval

The protocols used for the experimental animal studies was ratified by the committee in charge of animal ethics of the Animal Research Ethics Committee of the Evidence Science Research Institute of China University of Political Science and Law approved the study (# 2019012). In addition, all animal experimental protocols adhered to the American Veterinary Medical Association (AVMA) Guidelines for the Euthanasia of Animals (2020) guidelines. This current study also adheres to the ARRIVE Guidelines for reporting in vivo experiments.

## Results

### Successful building of the PTE rat model

**Figure 1:**
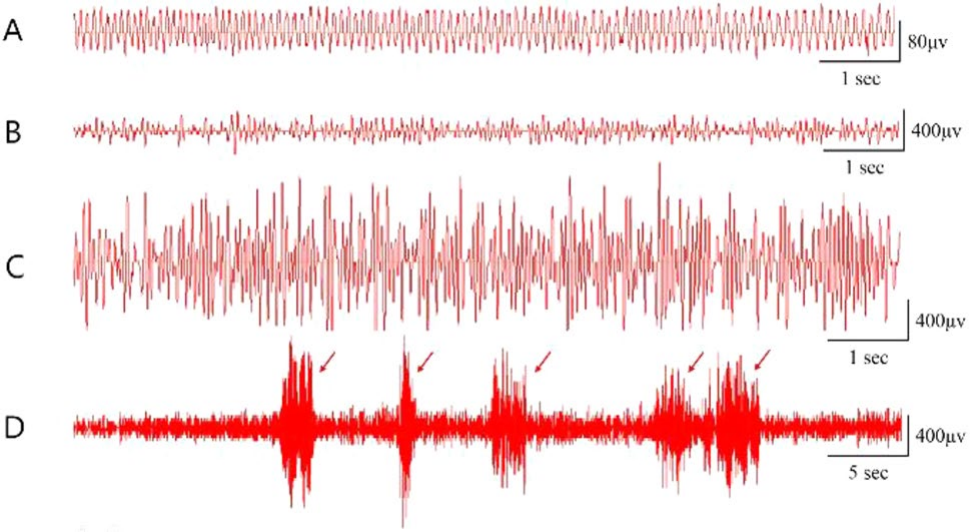
EEG monitoring results in PTE rats. Representative EEGs are shown. (**A**) Normal EEG at 60 min before injection; (**B**) multiple spike waves combined with slow waves; (**C**) two-phase and three-phase sharp waves; (**D**) intermittent abnormal discharge.

Behavioural changes and EEG results in rats were monitored and recorded to ensure the successful modelling of PTE rats. As shown in Fig. 1A–D, rats in the PTE group showed a significantly increased spike-wave. These were mainly composed of single spike waves, multiple spike waves, multiphase spike waves, two-phase spike waves, spike-wave rhythms, slow waves, slow spike waves, slow-wave combined with spike-wave rhythms, sharp waves, paroxysmal rhythm waves, and intermittent abnormal discharge compared with those in the sham group (*P* < 0.05). Also, compared with the sham rats, the PTE rats had behavioral seizures, which repeatedly occurred within 3 days and subsequently declined and were regularly developed at approximately 15 days post FeCl_2_ injection, all of which were consistent with our previous studies. The PTE group’s total Racine score was 810 (detailed information can be availed from the supplementary material of the previous study by our group)^12^. All these manifestations suggested the successful modelling of PTE rats.

### Identification of DEGs in PTE rats

**Figure 2:**
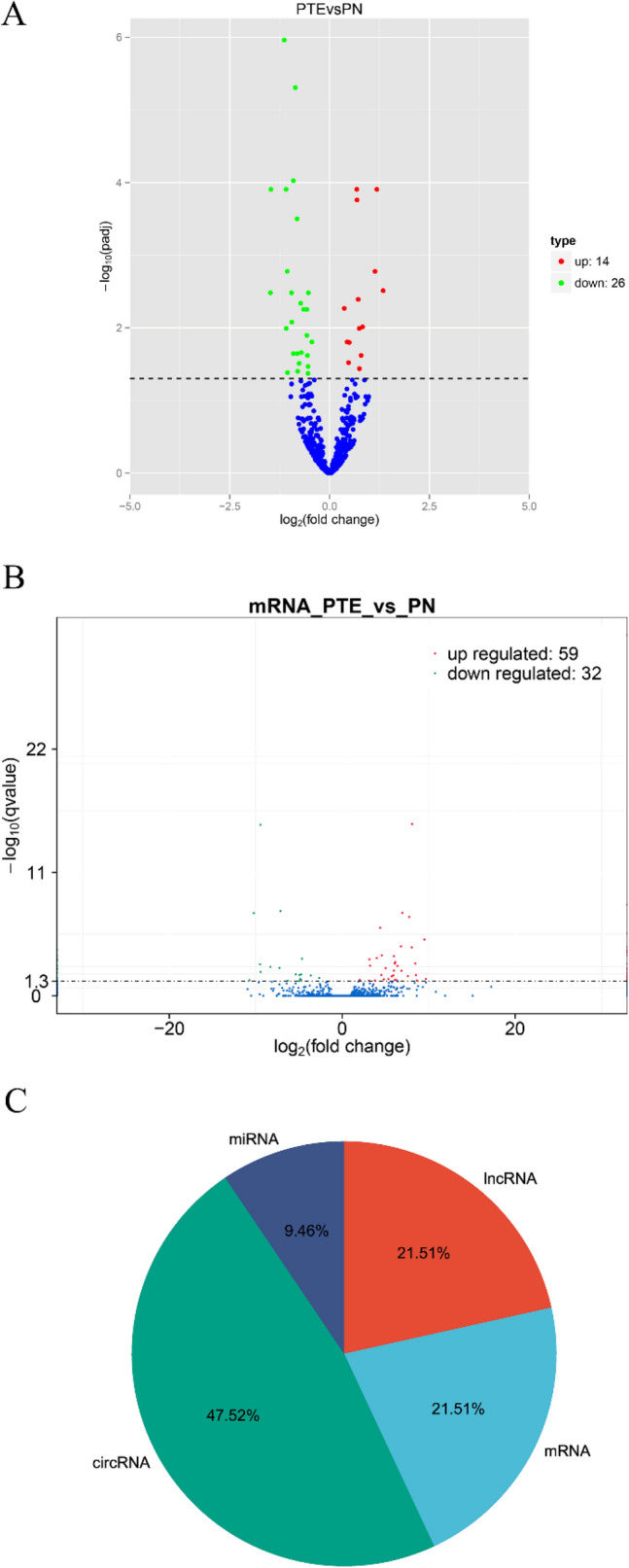
The analysis of whole transcriptome sequencing of brain tissues of PTE rats. Those in red are upregulated genes, and those in green are downregulated genes. Values shown log_2_ (fold changes) in the horizontal axis are the values of DEGs after logarithm. The values shown in the longitudinal axis log_10_ (*P*adj/qvalue) are negative values of *P* after the logarithm of 10. (**A**) The volcano plot shows the significantly differentially expressed miRNAs in PTE (n = 6). (**B**) The volcano plot shows the significantly differentially expressed mRNAs in PTE (n = 6). (**C**) Pie Chart shows the types and proportions of differentially expressed RNAs in PTE.

**Figure 3:**
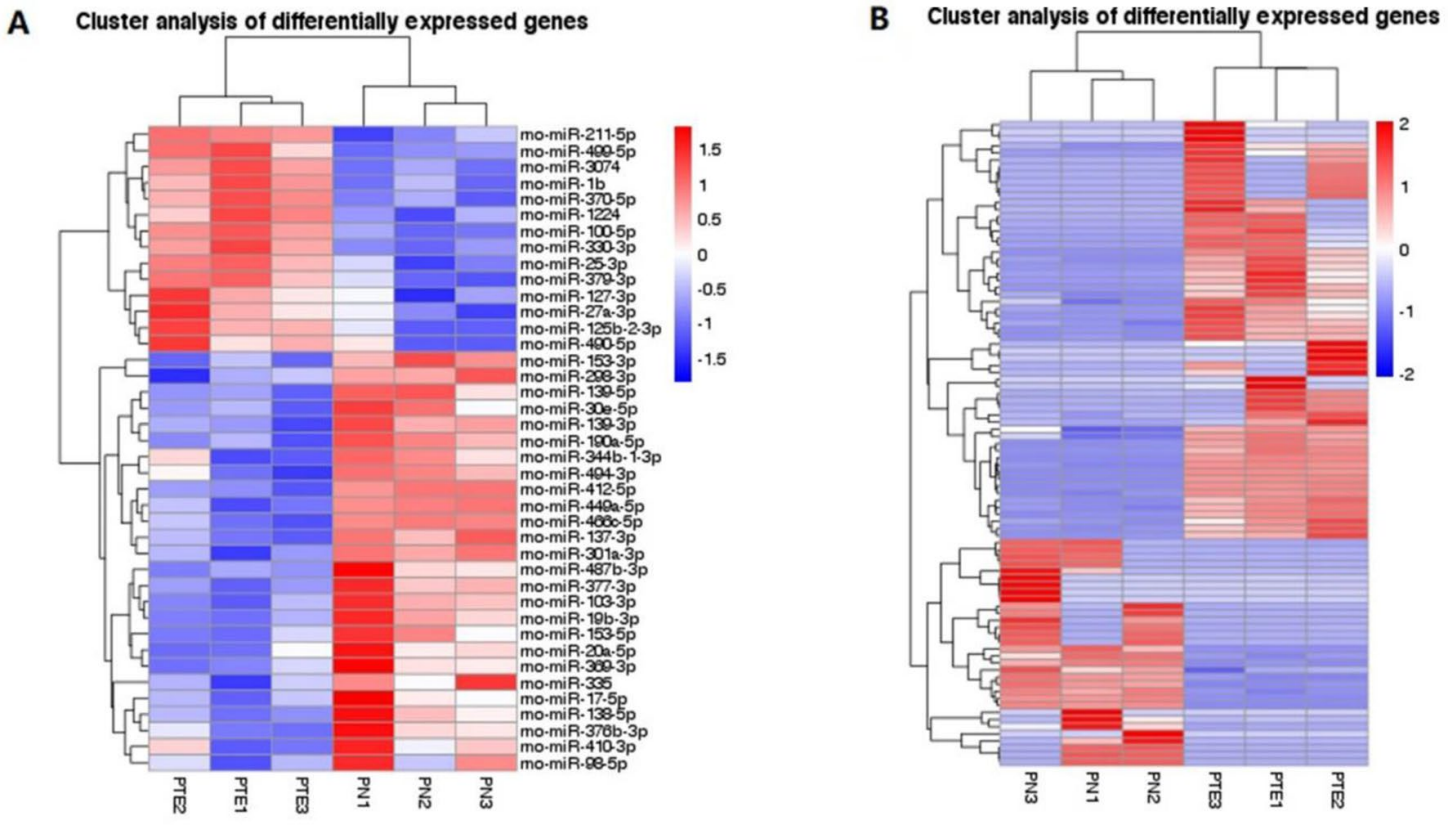
The heat maps of significantly differentially expressed miRNAs and mRNA in PTE rats. Each row represents one miRNA/mRNA. Each column represents samples from the PTE group and the control group. log_2_ (FC), the differential expression value after the logarithm of the expression difference multiple, was calculated. Red areas in the figure are expressions above the median of the control group, and blue areas are expressions below the median of the control group. (**A**) Heat map shows visually significantly differentially expressed miRNAs in PTE and the clustering analysis (n = 6). (**B**) Heat Map shows significantly differentially expressed mRNAs in PTE and the clustering analysis (n = 6).

There were 431 DEGs in the PTE group, including mRNA, miRNA, circRNA, and lncRNA, compared with the sham group. Therein, there were 40 differentially expressed miRNAs, including 14 upregulated ones and 26 downregulated ones (Fig. 2A), and 91 differentially expressed mRNAs, including 59 upregulated ones and 32 downregulated ones (Fig. 2B). miRNAs accounted for 9.28% among all DEGs in PTE, and mRNAs accounted for 21.11% (Fig. 2C). These findings suggested the presence of multiple DEGs during the onset of epilepsy in PTE rats. MiRNA expression profiling (Fig. 3A) and mRNA expression profiling (Fig. 3B) were drawn according to the hierarchical clustering analysis results of all differentially expressed miRNAs and mRNAs.

### Identification of calcium channel-related mRNAs in PTE

**Figure 4:**
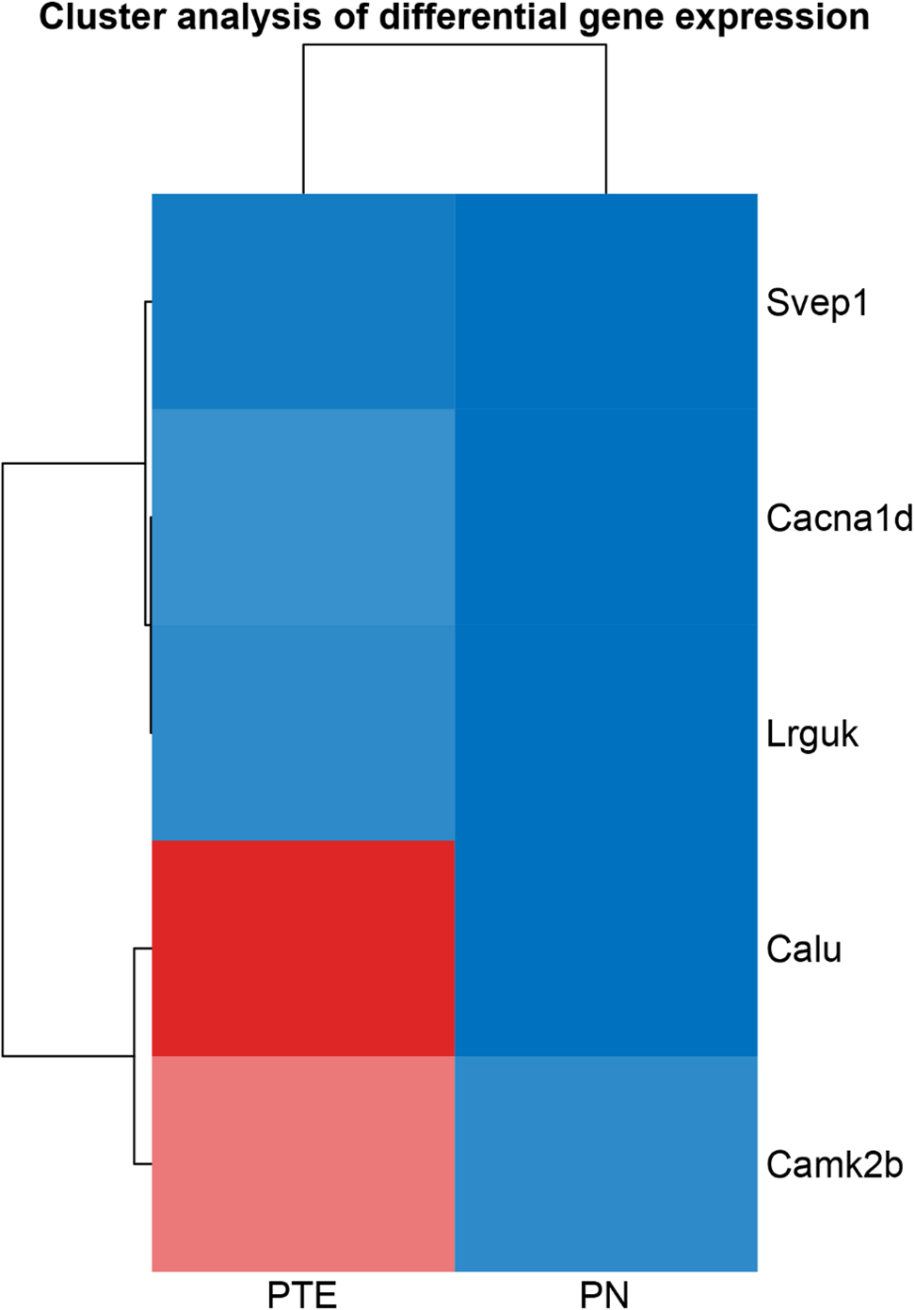
Visually differentially expressed mRNAs related to calcium ion signals in PTE and the clustering analysis.

Calcium channel-related mRNAs were screened out for hierarchical clustering analysis to draw its expression profiling in PTE. Among 91 differentially expressed mRNAs in PTE, five were related to the calcium channels, of which CALU, CAMK2B, LRGUK, CACNA1D, and SVEP1 were upregulated in PTE rats (Fig. 4). Their possible roles in calcium channels are described in Table 3. The CACNA1D gene belongs to the family of voltage-gated L-type Ca^2+^ channels. LRGUK has the structure of L-type calcium channel beta subunit Guanylate kinase-like domain. CALU belongs to a family of multiple EF-hand proteins and is a calcium-binding protein. CAMK2B belongs to the serine/threonine protein kinase family and the Ca^2+^/calmodulin-dependent protein kinase subfamily. SVEP1 has the structure of the EGF-like calcium-binding domain.

**Table 3:** Deferential mRNAs and functional annotations related to the calcium channel.

Gene symbol	Gene description
CALU	Calumenin kEF-hand domain pairk EF-hand Domain kEF-Hand 1, calcium-binding site
CAMK2B	Calcium/calmodulin-dependent/calcium dependent protein kinase Calcium/calmodulin-dependent protein kinase II, association-domain Protein kinase, ATP binding site NTF2-like domain Protein kinase-like domain Protein kinase domain Serine/threonine-protein kinase, the active site
LRGUK	Leucine-rich repeat domain, L domain-like-loop containing nucleoside triphosphate hydrolase Guanylate kinase/L-type calcium channel beta subunit Guanylate kinase-like domain leucine-rich repeat, typical subtype Leucine-rich repeat
CACNA1D	Voltage-gated calcium channel subunit alpha, C-terminal voltage-dependent channel, four helixes bundle domain Voltage-dependent L-type calcium channel, IQ-associated domain Voltage-dependent calcium channel, alpha-1 subunit Voltage-dependent calcium channel, alpha-1 subunit, IQ domain on transport domain Voltage-dependent calcium channel, L-type, alpha-1 subunit Voltage-dependent calcium channel, L-type, alpha-1D subunit
SVEP1	Tyrosine-protein kinase ephrin type A/B receptor-like EGF-like, conserved site Sushi/SCR/CCP domain EGF-type aspartate/asparagine hydroxylation site Concanavalin A-like lectin/glucanase domain EGF-like domain EGF-like calcium-binding domain Growth factor receptor cysteine-rich domain EGF-like calcium-binding, conserved site Pentaxin-related von Willebrand factor, type A Green fluorescent protein-likely domain

### Building of calcium channels-related miRNA–mRNA network in PTE

**Figure 5:**
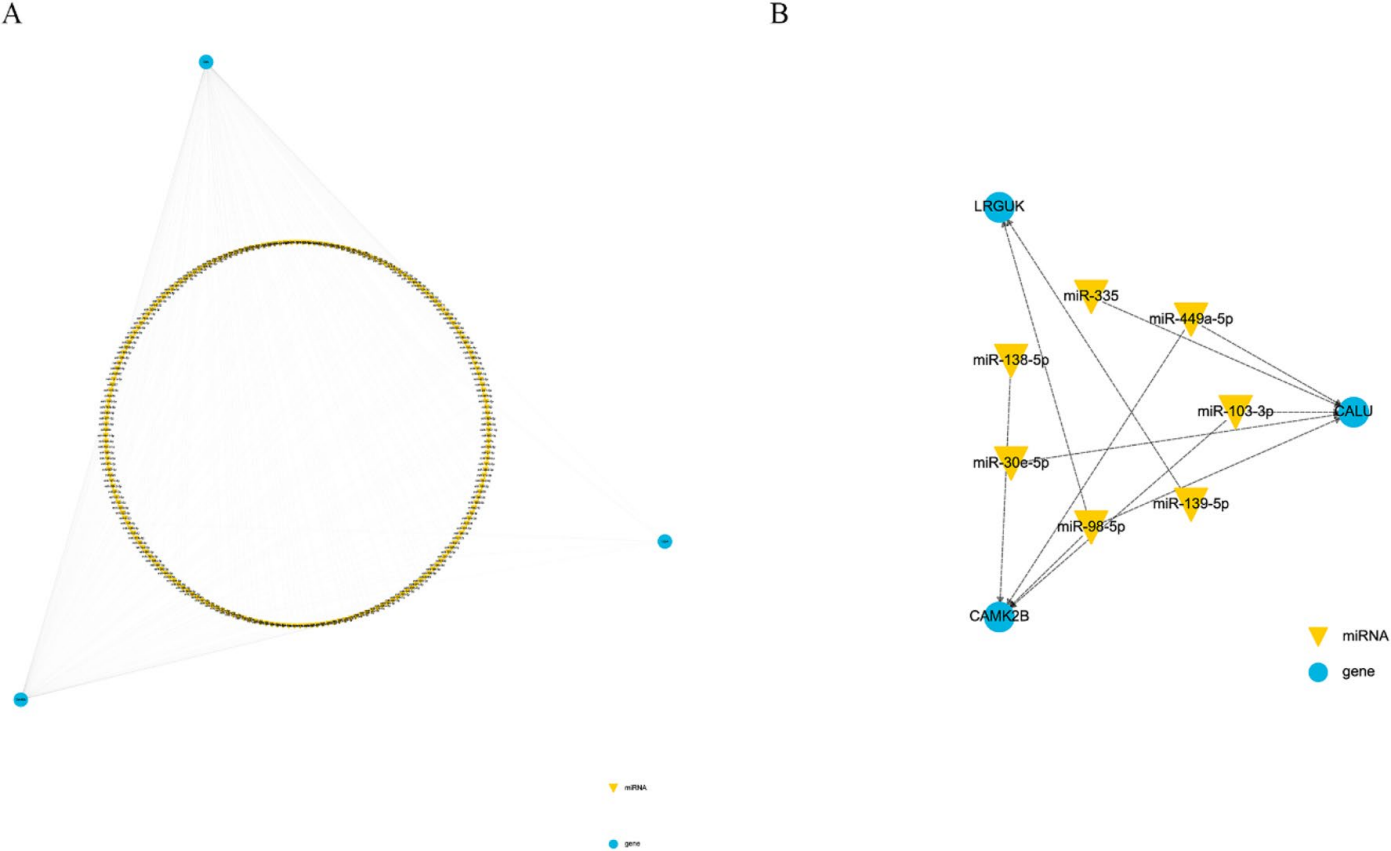
The calcium channel-related mRNA–miRNA network with PTE. The inverted triangle represents miRNA, and the circle represents mRNA. (**A**) A calcium channels-related miRNA–mRNA regulatory network in PTE centring on differentially expressed mRNAs. (**B**) A negative calcium channel-related miRNA–mRNA regulatory network based on comparison with the miRNA expression profiling in PTE.

A calcium channels-related miRNA–mRNA regulatory network in PTE centering on differentially expressed mRNAs was built (Fig. 5A). Whereafter, a negative calcium channel-related miRNA–mRNA regulatory network in PTE was constructed compared to the miRNA expression profiling in PTE (Fig. 5B). Results showed that miR-103-3p, miR-30e-5p, miR-335, miR-449a-5p and miR-98-5p negatively regulated CALU, miR-103-3p, miR-138-5p, miR-449a-5p and miR-98-5p negatively regulated CAMK2B, miR-139-5p and miR-98-5p negatively regulated LRGUK. While CACNA1D and SVEP1 did not form miRNA–mRNA pairs. In summary, our results indicate that five mRNAs and 7 miRNAs are associated with calcium channels in PTE and that there are 11 negatively correlated mRNA–miRNA pairs in PTE calcium channels.​

### Functional enrichment and pathway analysis of the DEMs

**Figure 6:**
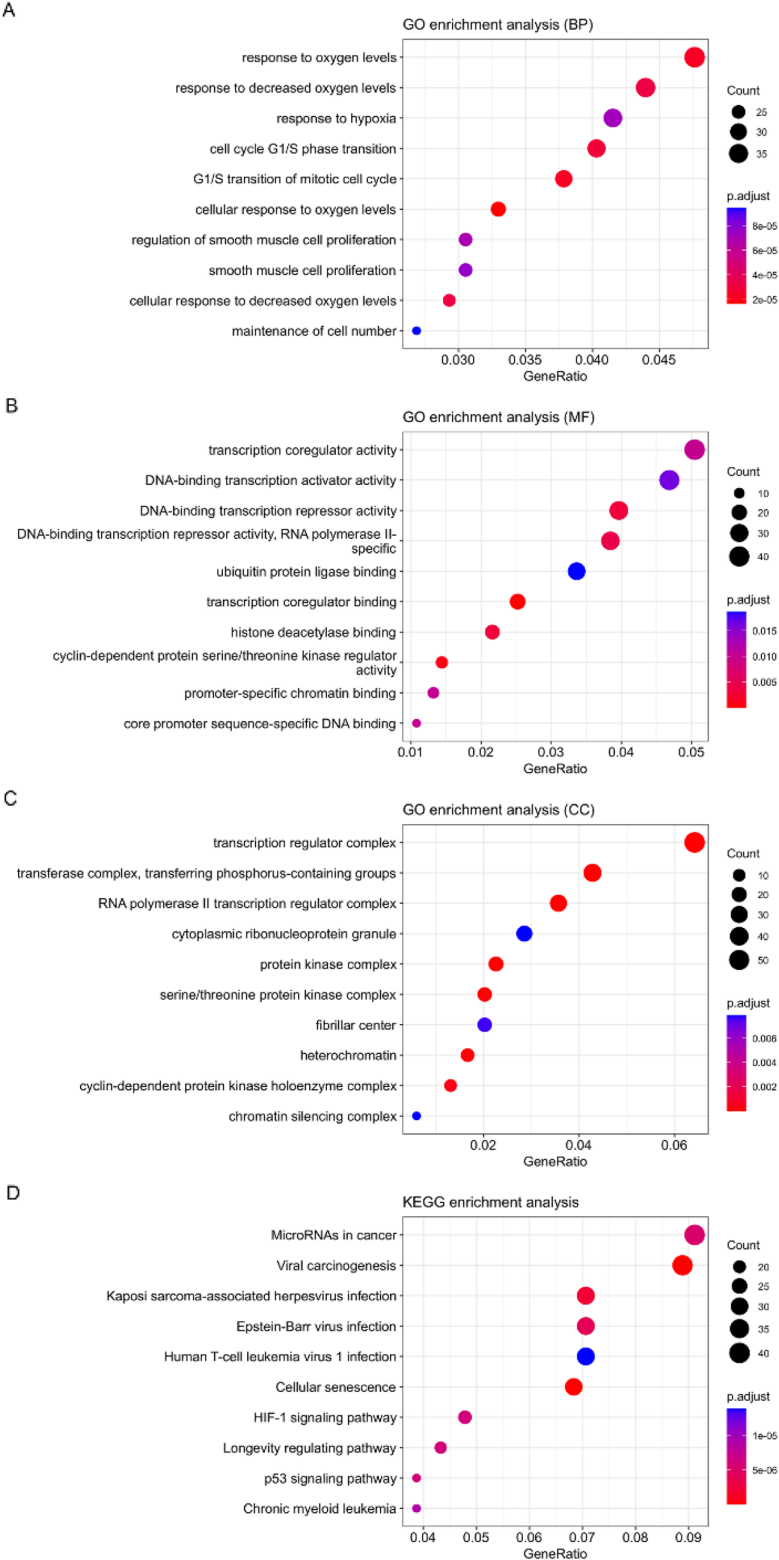
The GO annotation analysis and the KEGG pathway analysis of DEMs related to calcium ion signals in PTE. miRNAs were classified into three functional categories. 10 biological process annotations (**A**), 10 molecular function annotations (**B**), and 10 cellular component annotations (**C**) and the top 10 Kyoto Encyclopedia of Genes and Genomes pathways (**D**) were shown. *P* < 0.05.

The 7 DEMs were obtained by intersecting the results of the PTE differentially expressed miRNAs, with the miRNAs obtained from the calcium channel-associated mRNA prediction construct. In order to confirm the function of calcium channels related to DEMs, we performed GO and KEGG enrichment analyses. Figure 6 showed that 7 DEMs were enriched in biological processes, molecular function, and cellular components. The DEMs were classified into biological process annotations, such as cellular response to oxygen levels, response to oxygen levels, G1/S transition of mitotic cell cycle, response to decreased oxygen levels, regulation of smooth muscle cell proliferation, response to hypoxia, smooth muscle cell proliferation, maintenance of cell number (Fig. 6A); molecular function annotations, such as transcription coregulator binding, cyclin-dependent protein serine/threonine kinase regulator activity, DNA-binding transcription repressor activity, histone deacetylase binding, DNA-binding transcription repressor activity, RNA polymerase II-specific, transcription coregulator activity, core promoter sequence-specific DNA binding, promoter-specific chromatin binding, DNA-binding transcription activator activity, ubiquitin protein ligase binding (Fig. 6B); and cellular component annotations, such as transcription regulator complex, transferring phosphorus-containing groups, RNA polymerase II transcription regulator complex, protein kinase complex, serine/threonine protein kinase complex, heterochromatin, cyclin-dependent protein kinase holoenzyme complex, fibrillar center, cytoplasmic ribonucleoprotein granule, chromatin silencing complex (Fig. 6C). KEGG analysis showed that these miRNAs were relevant to the microRNAs in cancer, longevity regulating pathway, p53 signaling pathway, HIF-1 signaling pathway, chronic myeloid leukemia, human T-cell leukemia virus 1 infection (Fig. 6D).

### Verification of quantitative changes by RT-qPCR

**Figure 7:**
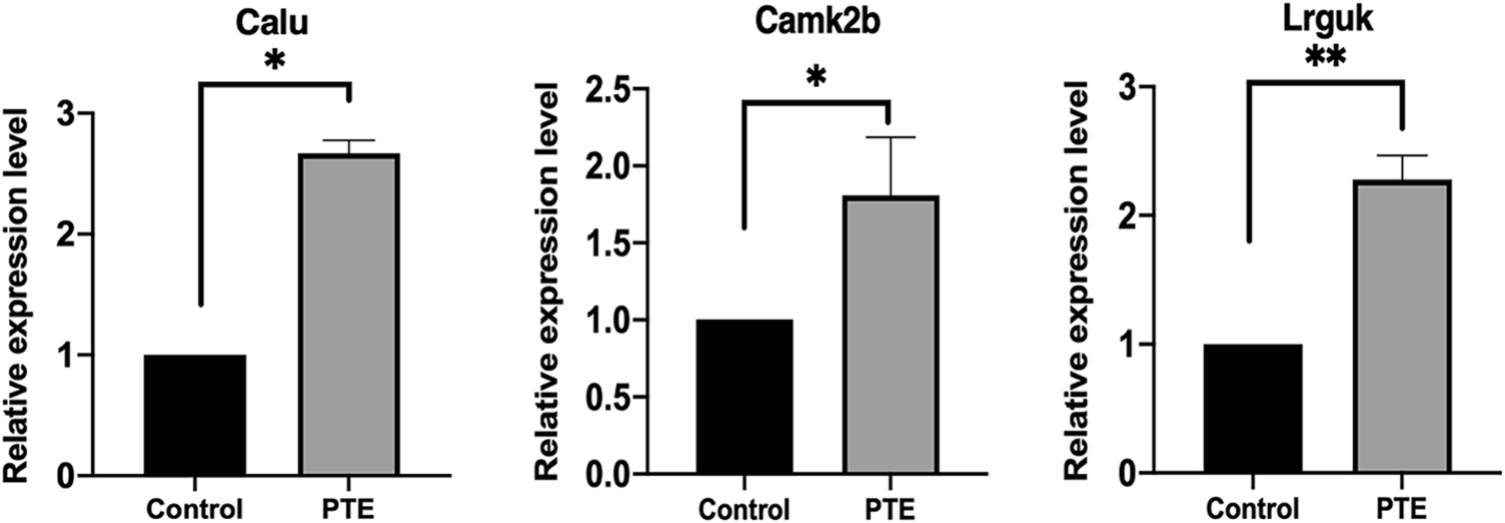
Real-time RT-PCR verification experiments of mRNAs. The expression of verified mRNAs (CALU, CAMK2B, LRGUK) was significantly increased in the PTE group compared with the sham group. *P < 0.05, **P < 0.01, ***P < 0.001.

**Figure 8:**
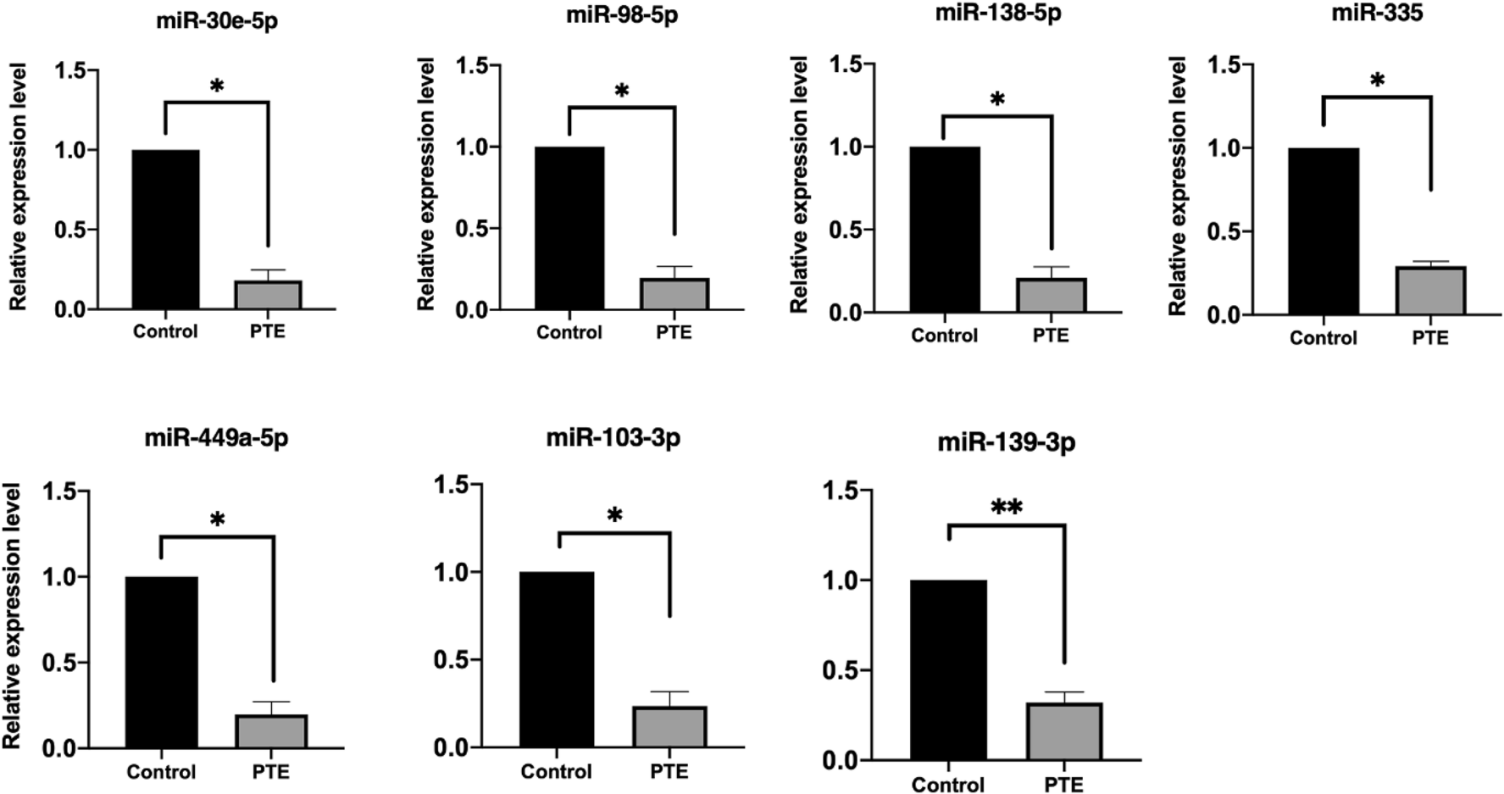
Real-time RT-PCR verification experiments of single miRNAs. The expression of verified miRNAs (miR-30e-5p, miR-98-5p, miR-138-5p, miR-335, miR-449a-5p, miR-103-3p, miR-139-3p) was significantly decreased in PTE group compared with the sham group. *P < 0.05, **P < 0.01, ***P < 0.001.

In order to verify the above RNA-seq result, 3 mRNAs and 7 miRNAs were chosen to be tested by RT-qPCR. Results showed that CALU, CAMK2B, and LRGUK were upregulated (Fig. 7). miR-30e-5p, miR-98-5p, miR-138-5p, miR-301a-3p, miR-335, miR-449a-5p, miR-103-3p, miR-139-3p were downregulated in PTE rats compared to the sham ones (Fig. 8). All the verified DEMs were negatively related to the differentially expressed mRNAs associated with calcium channels. Additionally, some DEMs were also consistent with our previous studies^12,14^. Together, the RT-qPCR results were in line with our RNA-seq results.

## Discussion

This study identified significantly dysregulated calcium channel-related mRNAs in the PTE rat brain. Among them, Cav1.3 belongs to the family of voltage-gated L-type Ca^2+^ channels and is encoded by the CACNA1D gene^16^. LRGUK has the structure of L-type calcium channel beta subunitkGuanylate kinase-like domain. CALU belongs to a family of multiple EF-hand proteins and is a calcium-binding protein. CAMK2B belongs to the serine/threonine protein kinase family and to the Ca^2+^/calmodulin-dependent protein kinase subfamily. SVEP1 has the structure of the EGF-like calcium-binding Domain.

Combined with the calcium channels-related miRNA–mRNA network, 7 miRNAs were involved with CALU, CAMK2B and LRGUK, forming 11 pairs of negatively correlated miRNA–mRNA pairs. Among them, previous research about LRGUK was focused on spermatogenesis and male fertility^17^, and no related studies on miRNA-LRGUK regulation were reported. Thus, we had an in-depth discussion only about CALU, CAMK2B.

CALU, encoding reticular calcium-binding proteins, is a low-affinity calcium-binding protein^18^. It is found at extremely low concentrations in the brain^19,20^, which is consistent with our results. Also, the expression of CALU is upregulated in PTE, which might directly regulate calcium channels by directly binding to Ca^2+^ through 7 EF-band structures^21^ or indirectly regulate calcium channels by manipulating the expressions of glutamic acid (Glu) and aspartic acid^22,23^. Correspondingly, the KEGG analysis of miRNA–mRNA pairs identified in this study (miR-103-3p/miR-30e-5p/miR-335/miR-449a-5p/miR-98-5p-CALU) indicated that these DEMs were related with the p53 signaling pathway, HIF-1 signaling pathway. The HIF-1 signaling pathway plays a crucial role in the entire process of neurodevelopment^24^. This could be closely related to the occurrence and development of epilepsy. Unfortunately, the role of CALU in PTE is still unclear and needs further elucidation.

CAMK2B effectively sensed the changes in the current strength of Ca^2+^ with its unique structure, thus regulating Ca^2+^ homeostasis^25^. At rest, CAMK2B was inactivated as a polymer^26^. At the same time, under transient Ca^2+^ current, it was activated after its autoregulatory region was bound to Ca^2+^ and calmodulin. Its autoinhibition was removed, and phosphorylation was enhanced. In the present study, the upregulation of CAMK2B might indicate its activation. CAMK2B might also regulate Ca^2+^ influx, which would initiate a series of rapid depolarization processes to make cells unusually excited, thus leading to epilepsy.

Besides all the mRNA and miRNA pairs, 7 DEMs related to the calcium channels were found to enrich the p53 signaling pathway and HIF-1 signaling pathway. Based on published literatures, miR-138, and miR-449a contribute to influencing the NF-κB signaling^27,28^. In addition, miR-335 and miR-138 are involved in the VEGF signaling pathway^29^. Besides, miR-377 could negatively regulate VEGFA to enhance angiogenesis and improve BBB permeability, whose dysfunction contributes to the epileptogenesis process^30,31^. In summary, the DEMs discovered in our study might involve in the occurrence and development of PTE.

## Limitations

The limitations of this study are: (1) the mechanisms of calcium channels are complex and we have not yet been able to elucidate the changes in the high-voltage and low-voltage activation systems of calcium channels in PTE. (2) The number of rats used in our study is relatively few. However, this was an initial study on this topic. We intend to increase the sample size in our future studies on this topic with extended experimental plans. (3) Verification experiments are lacking, and wet laboratory experiments are warranted to confirm these novel targets. For example, while our study found that miR-103-3p, miR-30e-5p, miR-335, miR-449a-5p and miR-98-5p negatively regulated CALU, miR-103-3p, miR-138-5p, miR-449a-5p and miR-98-5p negatively regulated CAMK2B, miR-139-5p and miR-98-5p negatively regulated LRGUK. However, we still need to confirm their regulatory relationship between PTEs through experimental approaches such as dual luciferase reporter genes, overexpression, and knockdown of miRNAs in cellular experiments as well as subsequent animal experiments, and it is worthwhile to continue exploring them in depth in future studies. (4) The animal's epileptiform activity quantification in detail was not performed in this study. The successful establishment of the PTE model was confirmed only by the Rachine score in EEG monitoring and the abnormal EEG discharges in the animal model. However, due to technical issues, 24-h continuous EEG recording for 30 days was not performed, we only monitored EEG data for part of the time. In a follow-up study, we also intend to extend the recording time of EEG for more details. We hope to investigate the frequency and size of seizures in relation to the transcriptomics of this model in a time-gradient manner, on the one hand, and in a time-series study, On the other hand, we hope to record not only the prolonged EEG and seizure frequency but also the details of the specific site of brain damage and the size of the damaged area in the animals by MRI and other methods to further investigate whether the seizures are correlated with the size and site of the damaged area. This warrants an in-depth investigation with a larger sample size. (5) The specificity of the EEG on PTE is not known, assuming it should be similar to epilepsy. For this reason, it is also not known how these changes in PTE would compare to a rodent with spontaneous Sprague–Dawley rats.

## Conclusion

In this study, five differentially expressed mRNA and 7 DEMs associated with calcium channels in PTE were identified. A total of 11 associated miRNA–mRNA pairs were identified and were critical for the pathogenesis of PTE. Unlike primary epilepsy, PTE is due to acquired epilepsy triggered by TBI. Therefore, a different biomarker from primary epilepsy may be a trauma-specific biomarker. Our current findings confirm that CALU is extremely low in the normal group, but increases significantly in the PTE group. However, it has not been reported in other types of epilepsy, and LRGUK has been very rarely studied in epilepsy. Therefore, CALU, LRGUK and their associated miRNAs may be specific biomarkers for PTE. Our study also found that miR-103-3p, miR-30e-5p, miR-335, miR-449a-5p and miR-98-5p negatively regulated CALU, miR-103-3p, miR-138-5p, miR-449a-5p and miR-98-5p negatively regulated CAMK2B, miR-139-5p and miR-98-5p negatively regulated LRGUK. However, our study only preliminarily explored the negative regulatory relationship between them, and future experiments such as overexpression, knockdown, and dual luciferase reporter genes in cells and animals are needed to further explore their regulatory mechanisms in PTE, which deserve to be explored subsequently.

## Data Availability

The data set for this study are available on the NCBI website, accessible with the following link: the Bioproject ID is PRJNA667324 (http://www.ncbi.nlm.nih.gov/bioproject/667324).
